# Molecular characterization and functional differentiation of three pheromone-binding proteins from *Tryporyza intacta*

**DOI:** 10.1038/s41598-018-29164-5

**Published:** 2018-07-17

**Authors:** Nainai Fang, Yuwei Hu, Bin Mao, Jie Bi, Ya Zheng, Chuxiong Guan, Yufeng Wang, Jihu Li, Yongkai Mao, Hui Ai

**Affiliations:** 1 Hubei Key Laboratory of Genetic Regulation and Integrative Biology, School of Life Sciences, Central China Normal University, Wuhan, 430079 China; 2 Guangdong Key Lab of Sugarcane Improvement & Biorefinery, Guangdong Provincial Bioengineering Institute (Guangzhou Sugarcane Industry Research Institute), Guangzhou, 510316 China

## Abstract

Insect pheromone-binding proteins (PBPs) have been proposed to capture and transport hydrophobic sex pheromone components emitted by con-specific insects to pheromone receptors in the hemolymph of male antennal sensilla. In this study, field trapping results indicate that a mixture of E11–16: Ald and Z11–16: Ald can effectively attract a great number of male *Tryporyza intacta*. Real-time PCR results suggest that the transcript levels of three *TintPBP1*-3 genes are mainly expressed in the adult antennae. Fluorescence competitive binding experiments show that TintPBP1-3 proteins have great binding affinities to their major sex pheromones. Moreover, TintPBPs clearly cannot bind to other four kinds of sex pheromone components released by another sugarcane borer, *Chilo venosatus* and *Chilo infuscatellu*, which have the same host plant and live in similar habitats like *T*. *intacta*. The molecular docking results demonstrate that six amino acid residues of the three TintPBPs are crucial for the specific perception of the sex pheromone components. These results will provide a foundation for the development of novel sex pheromone analogues and blocking agents for biological control of sugarcane pests, improving their efficient monitoring and integrated management strategies in the sugarcane field.

## Introduction

The insect antenna is a highly specific sensor and can discriminate exquisitely different odorant molecules including sex pheromones that stimulate insect behavioral responses^1^. The olfactory system of Lepidoptera is very sensitive to detect and differentiate similar sex pheromone compounds between the proximal species of insects, involving in the evolution of insect mating isolation and speciation^2–4^. In the field of insect olfactory research, there are many valuable model systems among moths to study the fundamental aspects of animal sensory perception at the molecular level^5,6^. In Lepidoptera, pheromone binding proteins (PBPs) were supposed to play their roles in sex pheromone perception mainly as pheromone carriers, by binding and transporting odorant molecules across the antennal hemolymph to the odorant receptor proteins^7,8^.

Insect PBPs are a class of small (16–18 kDa) soluble proteins containing six conserved cysteines^9^. The first member of the PBP family was discovered more than thirties years ago in the giant moth *Antheraea polyphemus* and was preferentially expressed in the male antennae^10^. Subsequently, a large number of Lepidoptera PBPs were identified and physiologically characterized from *Manduca sexta*^11^, *Lymantria dispar*^12^, *Antheraea polyphemus*^13^, *Spodoptera exigua*^14^, *Agrotis ipsilon*^15^ and *Sesamia inferens*^16^. For instance, Sun *et al*. reported that three PxylPBPs from the diamondback moth (*Plutella xyllotella*) not only robustly bound its four sex pheromone components but also significantly bound pheromone analogs^5^. HarmPBP1 from *Helicoverpa armigera* could also effectively bind to each of the two principal sex pheromones (Z-11-tetradecenal and Z-9-hexadecenal) of this pest^3^. Mao *et al*. found that MvitPBP3 not only has a high binding affinity with sex pheromones of *Maruca vitrata*, but also can bind several partial host-related semiochemicals from *Vigna unguiculata* and *Lablab purpureus*^17^. This demonstrates that PBPs may play multiple roles in sex pheromone perception of moths and host-plant recognition.

The sugarcane borer, *Tryporyza intacta* is one of the important sugarcane pests in Southeast Asian countries and South China, which has become particularly injurious in recent years^18^. It is an oligophagous insect species with a host range restricted to sugarcanes. In southern China, the early instars of *T*. *intacta* larvae can get into the stalks and cause serious harm to the sugarcane production^19^. Currently, a large number of conventional chemical insecticides are widely used to control this pest in sugarcane fields. However, due to its boring habit, the use of pesticides can generally result in residues and affect the quality of sugarcane. Therefore, integrated management strategies based on sex pheromones attraction have been developed as one important biological control techniques of agricultural pests. Previous study found that E11–16: Ald and Z11–16: Ald are the major sex pheromone components of *T*. *intacta*, which is useful for the population monitoring and mating disruption of this pest^20^. However, in addition to *T*. *intacta*, there are two other important pests, *Chilo venosatus* and *Chilo infuscatellus* in sugarcane, with different sex pheromone components. Sex pheromones of *C*. *venosatus* consists of a mixture of major components (Z13–18:AC, Z11–16:AC and Z13–18:OH) and only one sex pheromone component (Z11–16:OH) was identified from *C*. *infuscatellus*^21,22^. The present study will promote the understanding of olfactory molecular mechanism of *T*. *intacta* for discriminating six sex pheromone components released from *T*. *intacta*, *C*. *venosatus* and *C*. *infuscatellus*, improving the efficiency of semiochemical-based monitoring for this moth in the field.

## Materials and Methods

### Ethics Statement

The sugarcane borer, *T*. *intacta* larvae and adult moths were reared in our laboratory using artificial diet at 26 ± 1 °C (60 ± 10% RH and 14:10 h L: D). All experimental animal procedures including this pest were approved by the Institutional Review Board at Central China Normal University in China (CCNUIRB).

### Field trapping experiment

In the trapping experiment, the Custom-built Deltatraps with sticky inserts and rubber septa were purchased from Pherobio Technology Co. Ltd. and used in the field during the *T*. *intacta* flight season in 2016. Traps were suspended from iron stakes and placed approximately 25 m apart. 100 μl mixed sex pheromone solution (100 ng/μl) were prepared in hexane and added into rubber septa as lures, which the Hexane was used as blank control. The traps with three replicates were checked every day and the number of adult moths per trap was calculated for one week.

### Gene cloning and sequence analysis

Total RNA was extracted from the antennae of *T*. *intacta* and cDNA was synthesized according to the manufacturer’s instructions. The open reading frames (ORF) of *TintPBP*1 (Genebank: MF624766), *TintPBP2* (Genebank: MF624767) and *TintPBP*3 (Genebank: MF624768) genes were amplified by PCR method (Table 1). The PCR procedure was set during amplification phase of 30 cycles for 30 s at 94 °C, then 30 s at 60 °C followed by 45 s at 72 °C, and extend the chain at 72 °C for 10 min. The molecular weight of mature proteins were calculated with the ExPASy server program(http://web.expasy.org/compute_pi/) and the signal peptides were predicted by SignalP 4.0 (http://www.cbs.dtu.dk/services/SignalP/). The alignment of multiple sequences was conducted using Clustal X version 2.0 (http://cluster-x.org/). A phylogenetic tree was constructed using the MEGA version 6.0 neighbor-joining method with a p-distance model and pairwise gap deletion. Bootstrapping was performed to estimate the reliability of the branches using 1000 neighbor-joining replicates.

**Table 1:** Primers used in the experiments.

Primer name	Sequence (5′-3′)
PBP1-NcoF	CATGCCATGGCTTCCCAAGATGTTATGAAGCA
PBP1-XhoR	CCGCTCGAGTCAGACTTCTGCAAGCACC
PBP2-XhoF	CCGCTCGAGTCGCAAGACGTGATGAAAT
PBP2-EcoR	CCGGAATTCTTAGCCTTGCATATCAGC
PBP3-NcoF	CATGCCATGGCTGCAAATGTGAAAACAGATGAT
PBP3-XhoR	CCGCTCGAGTTACTCGTATTTGCTAATTTCT
PBP1YF	TCACGGGAATACAAAGGAGTT
PBP1YR	ACTTGCATACAGGAGTCGGTTT
PBP2YF	GATGCTGATACGGCAAAGAAAT
PBP2YR	CTGGAGCCCAATTGAGGTTA
PBP3YF	TTGAAATGGGTTACATAGACGC
PBP3YR	TTAGCGAAAATCATTGCTGTCC
ActinF	ATGATGAAATCGCCGCACTG
ActinR	CGACAATGGAGGGGAAGACA

### Expression patterns of *TintPBPs*

Real-time PCR experiments were performed to investigate the transcript levels of *TintPBPs* in different tissues of *T*. *intacta*. The experimental procedure was conducted in 25 mL reactions containing 2 μL of sample cDNA, 0.3 μL of each primer, 10 μL of 2× TransStart Top Green qPCR SuperMix and 7.4 μL of ddH_2_O. The quantitative real-time PCR used the following conditions: 95 °C for 3 min, followed by 40 cycles of 95 °C for 10 s and 50 °C for 30 s. Each sample was run with three technical replicates on three independent biological replicates. The 2^−ΔCt^ method was used to analyze the quantitative real-time PCR data.

### Expression and purification of *TintPBPs*

The pEASY-T1 Cloning Vector plasmid containing positive clones and pET32a/pET28a plasmid were digested with BamHI and XhoI restriction enzymes for 3 h at 37 °C. Target fragments were purified and ligated into a digested pET32a plasmid. The recombinant plasmids were transformed into DH5α *E*. *coli* competent cells and grown on LB solid medium with 10 mL ampicillin (50 mg/mL). Then the BL21 (DE3) Chemically Competent Cell (TransGen, Wuhan, China) were transformed with correct recombinant plasmids. After a single clone was collected and cultivated overnight in LB liquid medium, the culture was added into fresh medium (1:100) and cultured at 37 °C for 4 h. Protein expression was induced by the addition of IPTG (isopropyl-beta D-thiogalactopyranoside, 0.5 mM). Cells were grown for 4 h at 37 °C, and then the culture were harvested by centrifugation (10,000 rpm, 10 min). Subsequently, the suspension was crushed by sonication and then separated into supernatant and sediment by centrifugation. Then, the Ni ion affinity chromatography (Thermo, USA) was used to purify target proteins from the supernatant. The His-tag of TintPBPs proteins were removed by enterokinase and their purity were analyzed by SDS-PAGE.

### Fluorescence binding assays

To measure the affinities of the sex pheromones to TintPBP1-3 proteins, we used N-Phenyl-1-naphthylamine (1-NPN) as the fluorescent probe on a Hitachi F-4500 at 25 °C based on the method of Mao *et al*.^17^. 1-NPN was used as the fluorescent reporter (2 μM) and 0.5–10.0 μM for each competitor was used to test fluorescence competitive binding affinities of sex pheromones. Six compounds were selected and measured in competitive binding assays. The dissociation constant for 1-NPN and binding results were analyzed by Prism software. Each of IC_50_ values (concentrations of ligands halving the initial fluorescence value of 1-NPN) from competitors were calculated using following equation:

$$Ki=[{{\rm{IC}}}_{50}]/1+[1-{\rm{NPN}}]/{K}_{1-NPN},$$

where [1-NPN] is the free concentration of 1-NPN and *K*_*1*−*NPN*_ is the dissociation constant of the complex protein/1-NPN.

### Molecular docking

Three PBP proteins sequences were subsequently submitted to the SWISS-MODEL server (http://swissmodel.expasy.org/) for comparative structural modeling and displayed by PyMOL Viewer (http://www.pymol.org/). Position-Specific Iterated BLAST was used to search suitable templates for TintPBP1-3 proteins based on the RCSB Protein Data Bank (http://www.rcsb.org/pdb/home/home.do). The 3D model of pheromone binding proteins was built by homology modeling using the crystalline structure as the template. The optimum alignment was selected by the lowest Anolea score and the QMEAN4 score and the modeling rationality was further estimated using SAVE (http://services.mbi.ucla.edu/SAVES/). Based on the established homology model, we used the AutoDock Vina program to find the potential binding sites between the PBP proteins and ligands. The 3D structure of ligand was collected from ZINC (http://zinc.docking.org) and ChemBioOffice (version 14.0).

## Results

### Sequence analysis

**Figure 1:**
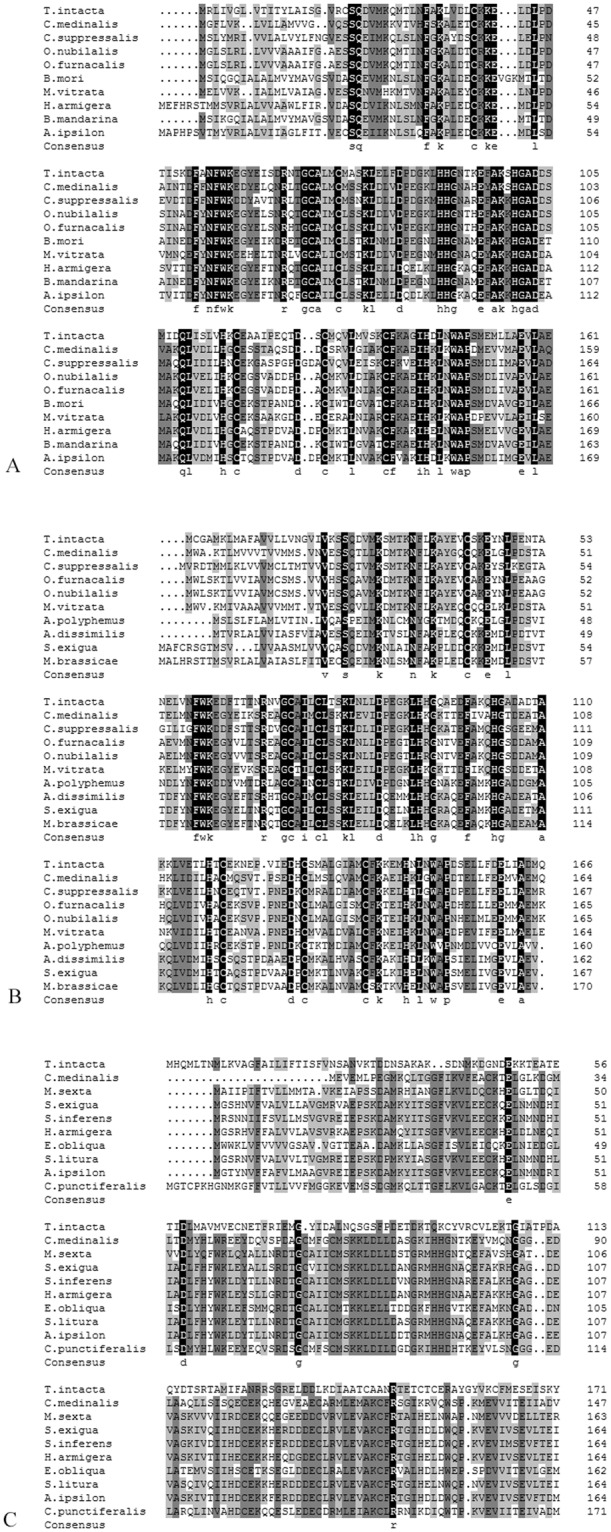
Multiple sequence alignment of TintPBPs with other Lepidopteran PBPs. (**A**) TintPBP1 is aligned with the PBP1 of other Lepidopteran moths including *Chilo suppressalis* (ACJ07123.1), *Ostrinia nubilalis* (ADT78495.1), *Ostrinia furnacalis* (ADT78500.1), *Bombyx mori* (AGR44764.1), *Maruca vitrata* (AGS46557.1), *Helicoverpa armigera* (AEB54585.1), *Bombyx mandarina* (ACT34881.1). (**B**) TintPBP2 is aligned with the PBP2 of other Lepidopteran moths including *Chilo suppressalis* (ADK66921.1), *Ostrinia furnacalis* (ADT78501.1), *Ostrinia nubilalis* (ADT78496.1), *Maruca vitrata* (AGS46555.1), *Antheraea polyphemus* (CAB86718.1), *Athetis dissimilis* (ALJ93809.1), *Spodoptera exigua* (AAU95537.1). (**C**) TintPBP3 is aligned with the PBP3 of other Lepidopteran moths including *Manduca sexta* (AAF16702.1), *Spodoptera exigua* (ACY78413.1), *Sesamia inferens* (AEQ. 30020.1), Helicoverpa armigera (AAO16091.1), *Ectropis obliqua* (ALS03849.1), *Spodoptera litura* (AKI87959.1), *Agrotis ipsilon* (AFM36758.1), *Conogethes punctiferalis* (ALC76551.1).

The three TintPBPs had the typical conserved six-cysteine signature and included signal peptides of 20, 23 and 26 amino acid residues, respectively, which are believed to form three disulfide bridges and the hydrophobic domains (Fig. 1). The predicted molecular weights of mature TintPBP1-3 proteins were 18.0 kDa, 18.6 kDa and 19.3 kDa, respectively. The calculated isoelectric points of mature TintPBP1-3 were 5.10, 5.18 and 4.72, respectively. The TintPBP1-3 proteins also shared high identities at amino acid level with previously identified PBPs in other lepidopteran species (Fig. 1). TintPBP1was similar to CmedPBP1 and OnubPBP1 with identity values of 52.57% and 59.43%, respectively, while TintPBP2 was similar to CsupPBP2 and OfurPBP2 with identity values of 61.05% and 57.56%, respectively. Besides, the amino acid sequence of the TintPBP3 exhibited very low similarity with other lepidopteran PBP3 proteins.

**Figure 2:**
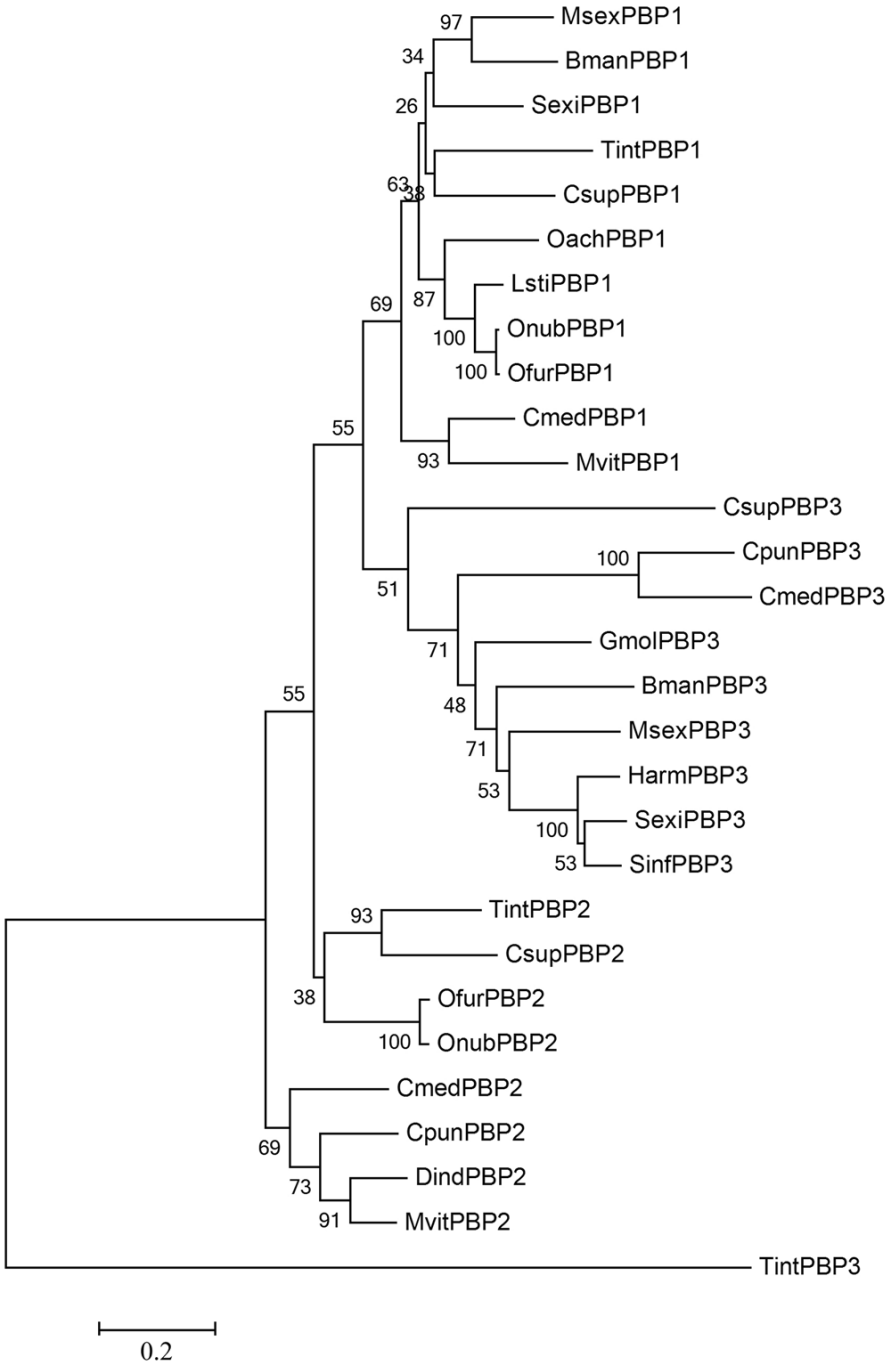
Phylogenetic tree of TintPBPs amino acid sequence with other Lepidopteran PBPs. The tree was constructed by the neighbor-joining method of MEGA (v6.0). GenBank accession numbers: CsupPBP1 (ACJ07123.1), SexiPBP1 (AAF06123.1), MsexPBP1 (AAA29326.1), BmanPBP1 (ACT34881.1), OachPBP1 (AEZ52490.1), LstiPBP1 (ACD67881.1), OnubPBP1 (ADT78495.1), OfurPBP1 (ADT78500.1), CmedPBP1 (AFG72997.1), MvitPBP1 (AGS46557.1), CsupPBP2(ADK66921.1), OfurPBP2 (ADT78501.1), OnubPBP2 (ADT78496.1), CmedPBP2(AGI37364.1), CpunPBP2 (ALC76550.1), DindPBP2 (BAG71419.1), MvitPBP2 (AGS46555.1), CsupPBP3 (ADL09140.1), CpunPBP3 (ALC76551.1), GmolPBP3 (AHZ89399.1), MsexPBP3 (AAF16702.1), HarmPBP3 (AAO16091.1), SexiPBP3 (ACY78413.1), SinfPBP3 (AEQ30020.1), BmanPBP3 (ACW84370.1).

The phylogenetic tree was constructed and used to assess the evolutionary relationships between the TintPBP1-3 protein sequences and other lepidopteran PBPs. As shown in Fig. 2, PBP1, PBP2 and PBP3 were respectively clustered each other in the phylogenetic tree, which was consistent with the highest sequence similarity among them. Multiple amino acid sequences alignment also suggested that PBPs had a high sequence similarity among diverse Lepidoptera species. Three PBP proteins were obviously separated from one another and were clustered to different subgroups (Fig. 2), highlighting that three *TintPBP1*-3 genes were also highly conserved in the olfactory genes family of Lepidoptera.

### Expression patterns of *TintPBPs*

**Figure 3:**
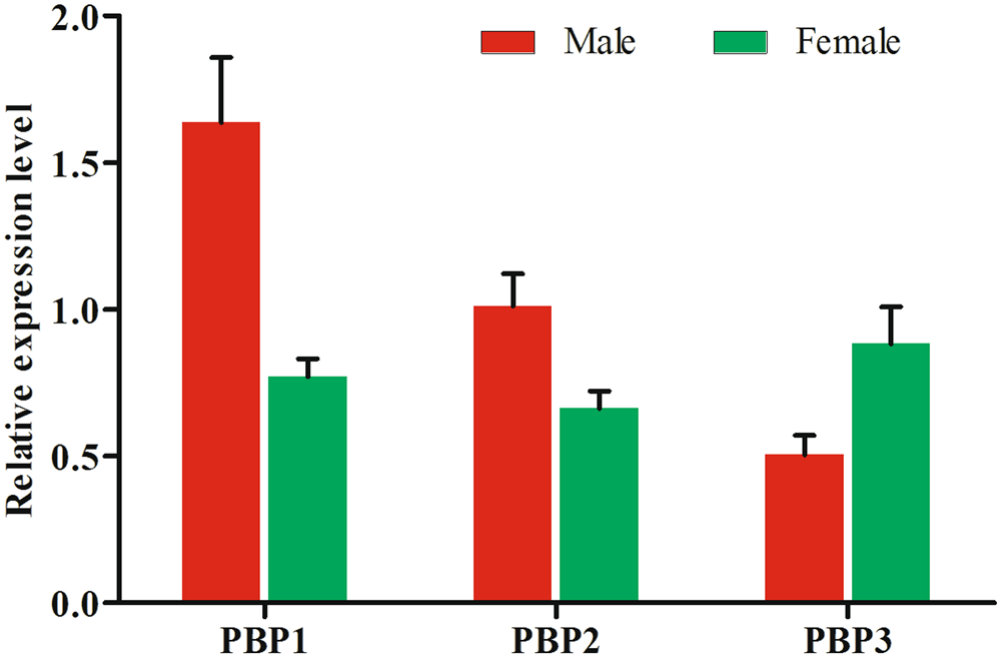
Relative transcript levels of TintPBPs in the adult antennae of *T*. *intacta*.

The transcript abundances of the *TintPBP1*-3 genes in the antenna were determined to understand the physiological functions of these PBP proteins. As shown in Fig. 3, three *TintPBP1*-3 genes were specifically expressed in the male and female antennae of adult moths at relatively high levels. Moreover, the expression level of these *TintPBP1*-*3* genes was sex-biased, which the *TintPBP1*-2 genes were highly expressed in the male antennae with 2.12-fold and 1.53-fold increases compared with that of females. In contrast, the transcript level of *TintPBP3* gene was female-biased with 1.75-fold increase compared with the antennae of male *T*. *intacta*.

### Expression and purification of recombinant TintPBPs

**Figure 4:**
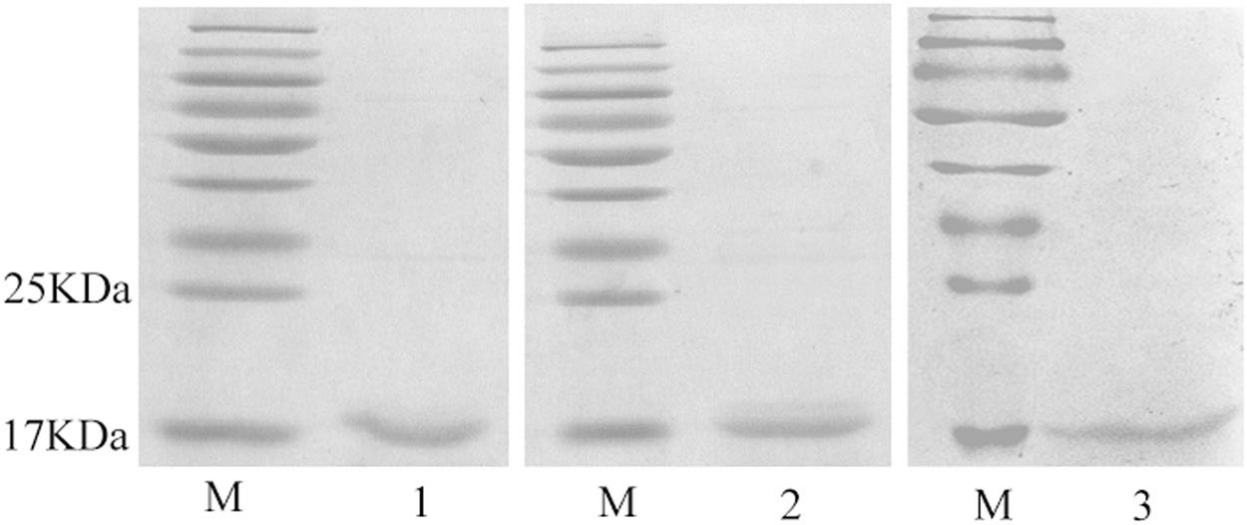
SDS-PAGE analysis of recombinant proteins. Lane 1 - Purified TintPBP1 without His tag, Lane 2 - Purified TintPBP2 without His tag, Lane 1 - Purified TintPBP3 without His tag, Lane M - Marker protein.

The recombinant plasmid pET32a(+)/PBP1, pET28a(+)/PBP2 and pET32a(+)/PBP3 were transferred into *E*. *coli* BL21 (DE3) competent cells. Three TintPBPs protein were soluble and purified with Ni-NTA resin in accordance with our previous reported protocols^17^. Moreover, to avoid a possible effect by the His-tag on subsequent experiments, this tag was removed by digestion with enterokinase (rEK). Finally, a second purification was carried out and a single band of predicted size was observed (Fig. 4).

### Field trapping experiments of sex pheromones and binding characterization of TintPBPs

**Figure 5:**
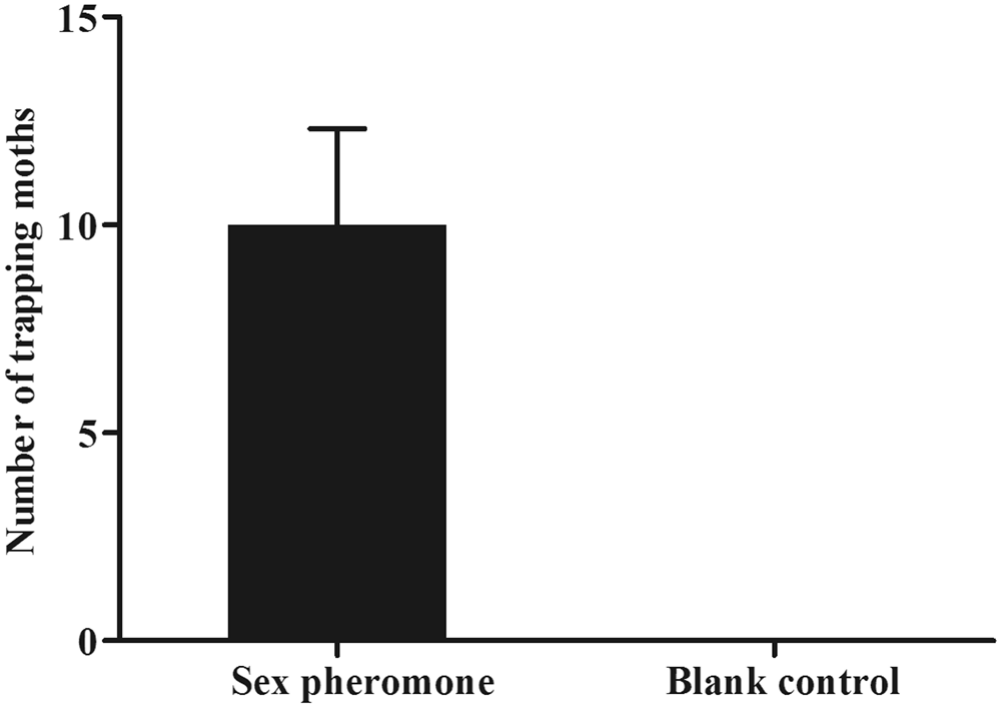
*T*. *intacta* adult moths caught in the field trapping experiments for one week.

**Figure 6:**
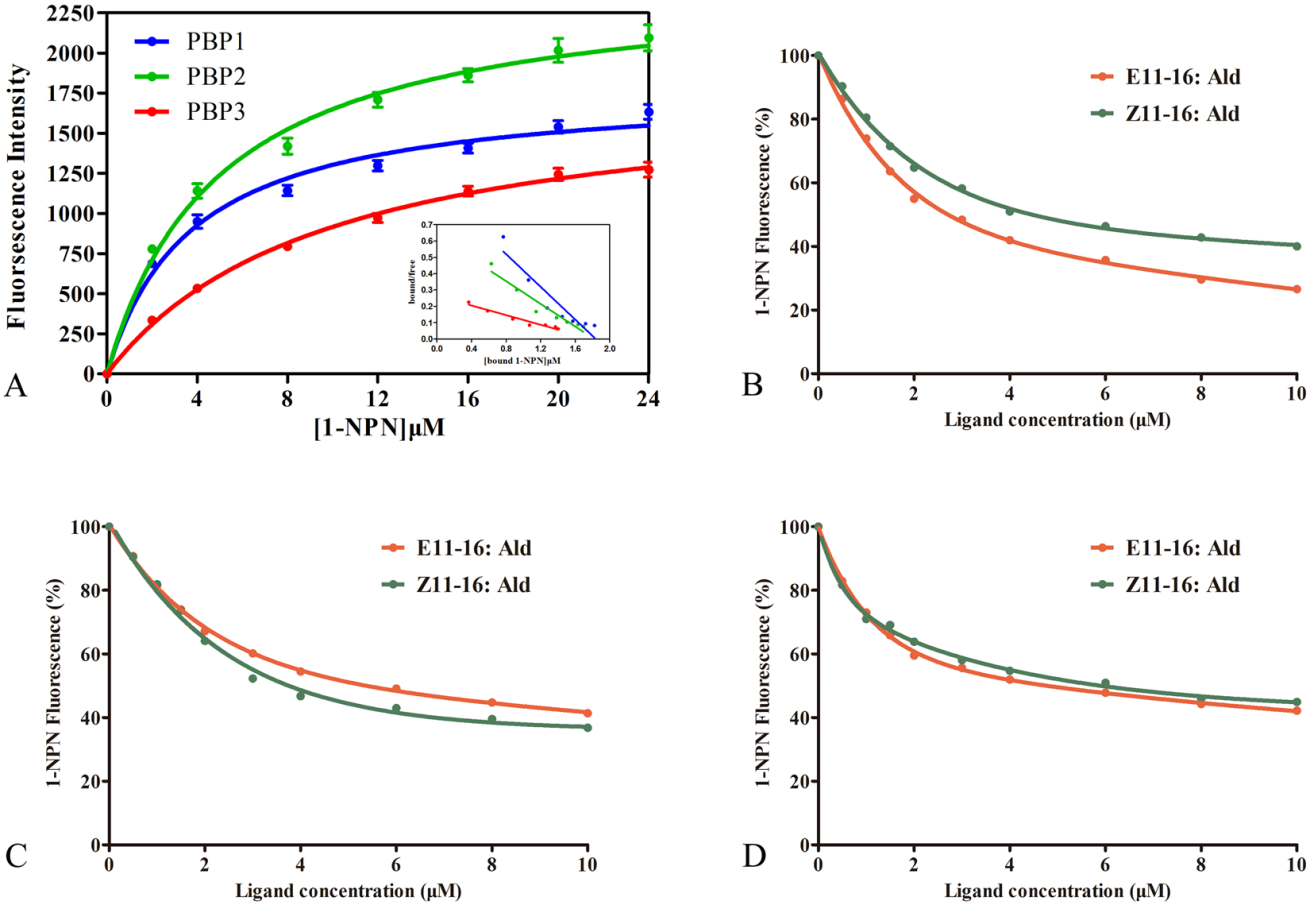
Ligand-binding experiments. (**A**) Binding curve and relative Scatchard plot. (**B**–**D**) Competitive binding curves of sex pheromone components from *T*. *intacta* to TintPBP1-3 proteins. (**E**–**G**) Competitive binding curves of four sex pheromones from *Chilo venosatus* and *Chilo infuscatellus* to TintPBP1-3 proteins.

**Figure 7:**
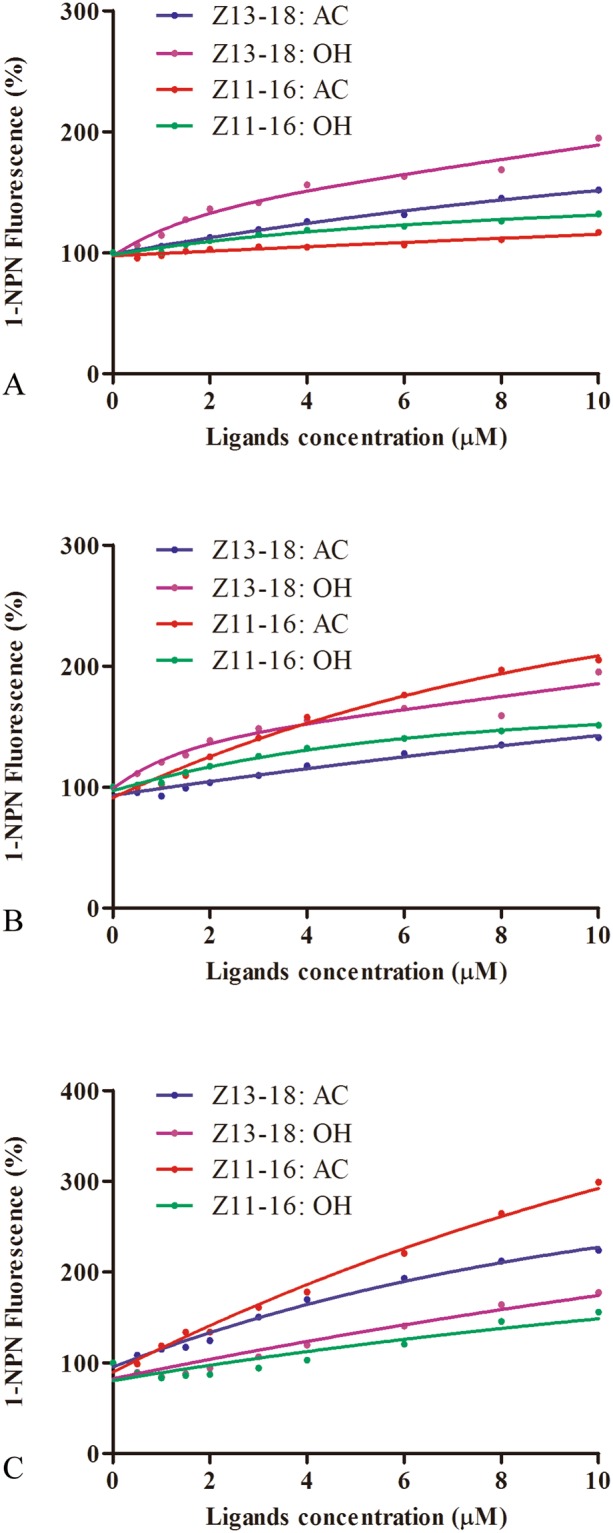
Ligand-binding experiments. (**A**–**C**) Competitive binding curves of four sex pheromones from *Chilo venosatus* and *Chilo infuscatellus* to TintPBP1-3 proteins.

The sex pheromones of *T*. *intacta*, E11–16: Ald and Z11–16: Ald were measured in the field trapping experiment. The trapping results suggested the sex pheromone lures could effectively attract the adult moths and showed significant difference compared with the blank lure (Fig. 5). Based on the trapping results, we investigated their ligand binding specificity to six kinds of volatile sex pheromones, including E11–16: Ald, Z11–16: Ald (*T*. *intacta*), Z13–18:AC, Z11–16:AC and Z13–18:OH (*C*. *venosatus*) and Z11–16:OH (*C*. *infuscatellus*). First, we examined the dissociation constants between the TintPBPs and the fluorescence probe 1-NPN, and the values for the dissociation constants ranged from 2.0 μM to 24.0 μM. The binding curves and Scatchard plots indicated that their great affinities of three TintPBPs and the 1-NPN, which increased linearly with concentration of the fluorescence probe (Fig. 6A). Present results revealed that different specificities among the three PBP proteins (Table 2). TintPBP1 was the most sensitive to E11–16: Ald, which its *Ki* value (the calculated inhibition constants) was 2.15 μM (Fig. 6B and Table 2). The TintPBP2 protein suggested the best binding capacity to Z11–16: Ald, with the *Ki* value of 2.83 μM. Besides, TintPBP3 also exhibited great binding capacity to both of volatile sex pheromones from *T*. *intacta*, with the *Ki* values of 3.45 μM and 3.96 μM, respectively (Fig. 6C,D). Additionally, as shown in Fig. 7A–C, the TintPBP1-3 proteins cannot bind any other sex pheromone components (Z13–18:AC, Z11–16:AC, Z13–18:OH and Z11–16:OH) from *C*. *venosatus* and *C*. *infuscatellus*.

**Table 2:** The binding constants of different ligands.

Compounds	TintPBP1		TintPBP2		TintPBP3	
	IC_50_ (μM)	*K*_i_ (μM)	IC_50_ (μM)	*K*_i_ (μM)	IC_50_ (μM)	*K*_i_ (μM)
E11–16: Ald	3.09 ± 0.22	2.15 ± 0.16	4.78 ± 0.17	3.58 ± 0.13	4.08 ± 0.24	3.45 ± 0.21
Z11–16: Ald	4.27 ± 0.05	2.95 ± 0.04	3.81 ± 0.15	2.83 ± 0.11	4.66 ± 0.33	3.96 ± 0.28

Binding of 1-NPN and different sex pheromone components to TintPBP1-3.

Note: IC_50_, ligand concentration displacing 50% of the fluorescence intensity of the TintPBPs/N-phenyl-1-naphthylamine complex; *Ki*, dissociation constant.

### Molecular docking

**Figure 8:**
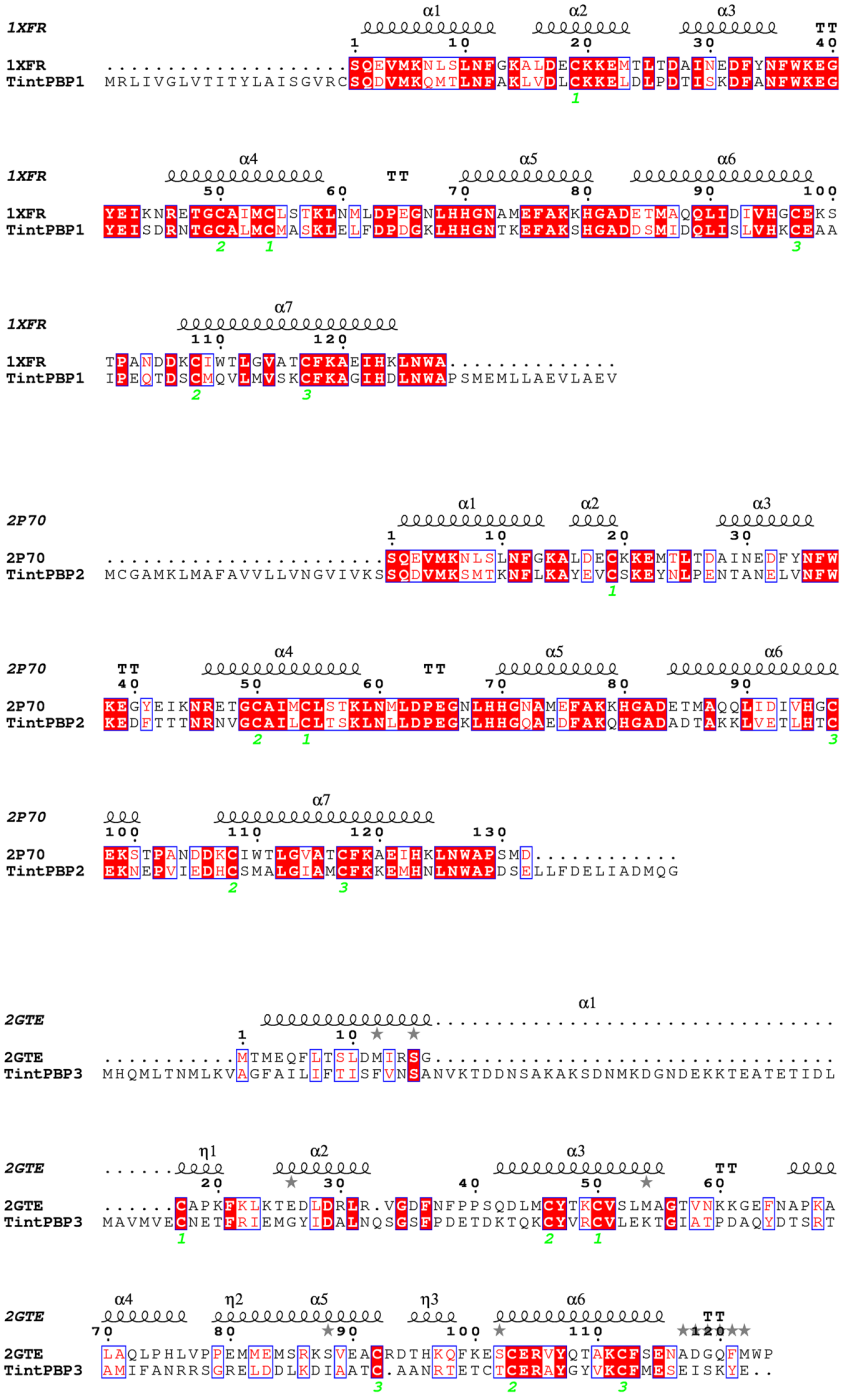
Sequence alignment of TintPBPs and templates. Conserved residues are highlighted in white letters with a red background. Six conserved residues are labeled by pentagram. The disulfide bridges are numbered 1 to 3. α-helices are displayed as squiggles.

**Figure 9:**
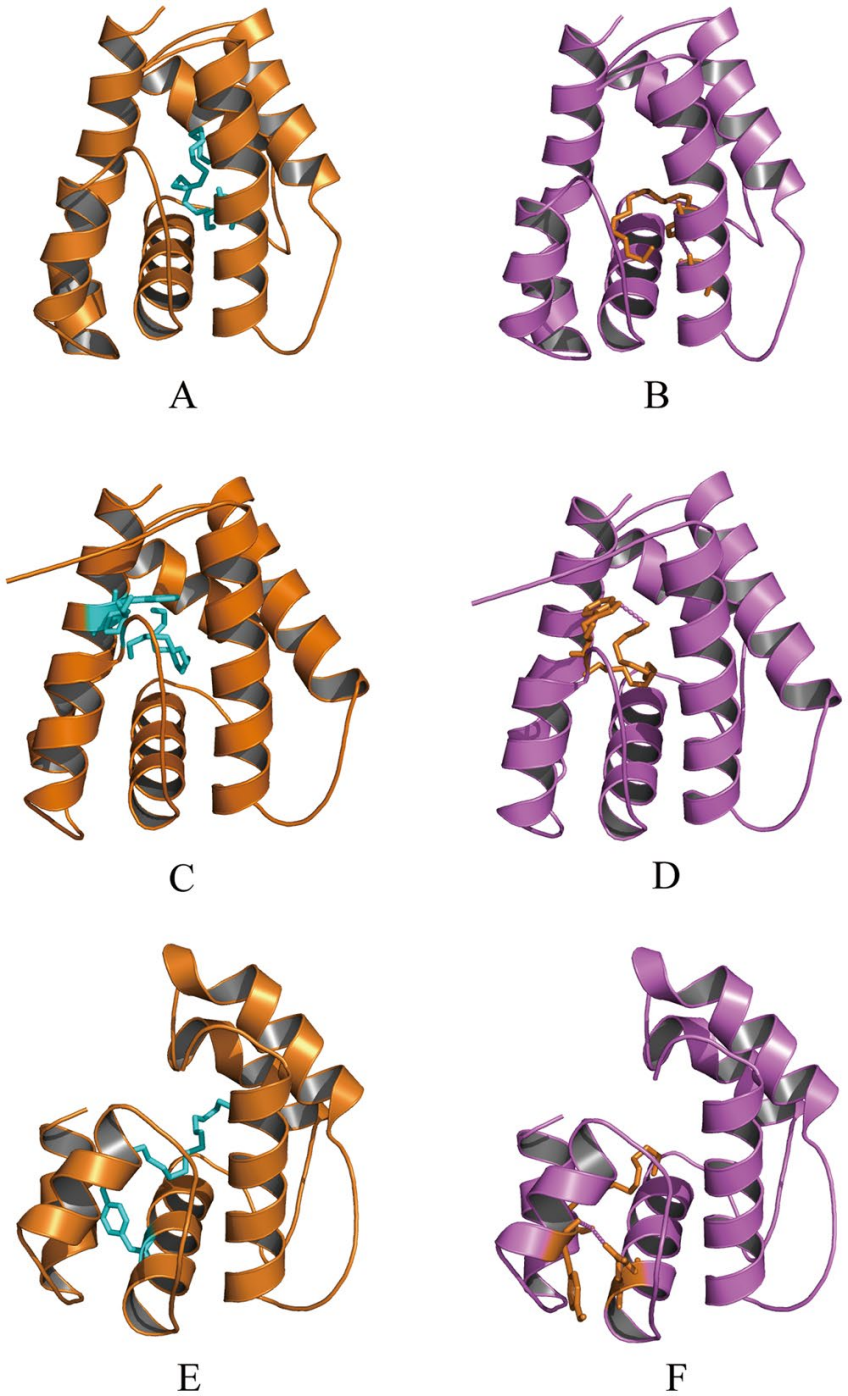
Molecular docking of TintPBPs and sex pheromone ligands. (**A**) E11–16: Ald; (**B**) Z11–16: Ald.

Molecular docking was used to analyze potential amino acid binding sites of TintPBPs with E11–16: Ald and Z11–16: Ald. As shown in Fig. 8, the amino acid sequences of TintPBP1-3 proteins were compared with three PBPs templates (PDB ID code: 1XFR, 2P70, 2GTE). After sequence alignment analysis, the 3D structures of TintPBP1-3 proteins were also constructed by SwissModel according to the crystal structures of templates. Subsequently, both of sex pheromone molecules from *T*. *intacta* were docked into the binding pockets of the three TintPBP proteins. As shown in Fig. 9, three 3D structures of TintPBPs were formed by a roughly conical arrangement of six α-helices connected by loops. The three PBP proteins exhibited the strong interactions to E11–16: Ald and Z11–16: Ald, which had the similar docking interaction energy values (−3.51, −5.00 and −5.11 kcal/mol, E11–16: Ald; −4.23, −4.82 and −3.30 kcal/mol, Z11–16: Ald) (Table 3). However, their key amino acid binding sites has obvious difference, such as serine 135 (S135) and valine 131 (V131) of TintPBP1, threonine 32 (T32) and tryptophan 60 (W60) of TintPBP2, tyrosine 98/77 (Y98, Y77) and glutamine 95 (Q95) of TintPBP3 (Fig. 9 and Table 3), involving in the formation of hydrogen bond between the TintPBPs and their sex pheromones.

**Table 3:** The docking results of TintPBP1-3 with different ligands.

Compounds	Cdocker interaction energy (Kcal/mol)			Residues forming H-bond with ligand		
	PBP1	PBP2	PBP3	PBP1	PBP2	PBP3
E11–16:Ald	−3.51	5.00	5.11	S135	T32/W60	Y98
Z11–16:Ald	−4.23	4.82	3.30	V131/S135	W60	Y77/Q95

## Discussion

The olfactory system of insects is essential for Lepidoptera as well as in other insect orders to initiate behavioral responses, such as searching for food sources, mating, oviposition and feeding^23^. The binding of PBPs, odorant binding proteins (OBPs) and chemosensory proteins (CSPs) with volatile compounds from the environmental stimuli is the first step for insects to identify odors and tastants, which they are important for their survival and reproduction^24,25^. PBPs are regarded at the beginning as passive carriers of sex pheromones across the antennal hemolymph to the odorant receptors of insect. *T*. *intacta* is a serious pantropical pest, and its sex pheromones have been used as the biological control agents in the sugarcane production of southern China^18,19^. Our field trapping results also show that E11–16: Ald and Z11–16: Ald can be used in the monitoring and forecasting of this pest, which is similar to other sex pheromones of Lepidoptera species. Actually, in addition to *T*. *intacta*, there are many species of borers, such as *C*. *venosatus* and *C*. *infuscatellus* in the sugarcane field, which may interfere with their sex pheromones recognition each other, thus affecting the biological prevention of these pests in the sugarcane field^21,22^. Therefore, the molecular characterization and binding properties of PBPs with major sex pheromones of *T*. *intacta* will help to understand the olfactory molecular mechanism of this sugarcane borer and provide further detailed evidences for the olfactory bait and interfering agents of this pest.

Qualitative real-time PCR analysis of these three *TintPBP* genes showed predominant expression in the antennae of adult moths, which was similar with many other lepidopteran species, such as *Spodoptera exigua*^14^, *Helicoverpa armigera*^3^, *Agrotis ipsilon*^15^, *Sesamia inferens*^16^ and *M*. *vitrata*^17^. Moreover, the transcript abundances of the *TintPBP* genes were sex-biased and exhibited obvious difference in the male and female antennae. For instance, the expression level of *TintPBP1*-2 genes in the male antennae was significantly higher than that of female moths, highlighting that *TintPBP1* and *TintPBP2* genes were mainly involved in olfactory recognition of sex pheromone released by the pheromone gland of female *T*. *intacta*. Mating behavior of Lepidoptera moths initiate by calling females releasing sex pheromones, and conspecific males in surrounding areas sense the pheromone and respond by flying toward the calling females^26^. Therefore, male-biased expression level suggests that the TintPBP1-2 proteins may play an essential role in the sexual communication and mating of *T*. *intacta*. Interestingly, *TintPBP3* gene was more abundantly expressed in the female antennae of *T*. *intacta* compared with that of male moths. Therefore, we speculated that TintPBP3 may be specially involved in the female autodetection to the sex pheromones, which has been demonstrated in other lepidopterans species, such as AipsPBP3 of *Agrotis ipsilon*^15^ and MvitPBP3 of *M*. *vitrata*^17^.

The insect PBPs are mainly involved in discrimination of conspecific and heterogenous species in the field through their binding characterization with different sex pheromone components and semiochemicals^12,27^. In this study, ligand binding specificity of PBPs with six kinds of sex pheromones from *T*. *intacta*, *C*. *venosatus* and *C*. *infuscatellus* are tested in the fluorescence competitive experiments. Both of the sex pheromone components of *T*. *intacta*, E11–16: Ald and Z11–16: Ald can strongly bind with three TintPBP1-3 proteins, with different levels of sensitivity. TintPBP1 and TintPBP2 proteins are the most sensitive to E11–16: Ald and Z11–16: Ald, respectively. Moreover, based on the 3D structural models and docking study, hydrogen bonds are the main linkage between TintPBP1-3 proteins and sex pheromone ligands. Hydrogen bonds have been confirmed as the primary linkage between proteins and ligands in several insect PBPs and OBPs^28–32^. Additionally, three TintPBP1-3 proteins cannot bind another four sex pheromones of *C*. *venosatus* and *C*. *infuscatellus*, which share the same host plant with this borer. These results indicated that *T*. *intacta* can easily distinguish different borers through the combination of TintPBP1-3 proteins and autologous sex pheromones, thus avoiding their mating disorders in the same sugarcane field. This method of olfactory recognition is very beneficial to the reproduction and systematic evolution of various insect species, which have the same host plant and live in the similar habitat. Therefore, present results may be clearly defined as olfactory molecular mechanism of *T*. *intacta* adult moths for easily discriminating different sex pheromone components, which released by three kinds of pests, *T*. *intacta*, *C*. *venosatus* and *C*. *infuscatellus*.

In conclusion, both of sex pheromone components of *T*. *intacta* can effectively attract a great number of adult moths. Ligands binding specificity also indicate that the TintPBP1, TintPBP2 and TintPBP3 are responsible for the recognition of the major sex pheromone component, E11–16: Ald and Z11–16: Ald. These findings may help clarify physiological roles of TintPBPs in the sex pheromone recognition pathway of *T*. *intacta*, which in turn can facilitate pest control by exploring sex pheromone blocking agents. Our research will also lead to the development and potential application of sex pheromone and their analogues for biological control of various sugarcane pests.
